# Kinetic and static perimetry after 16 years and additional OCT-A analysis in eyes with long-lasting optic disc drusen

**DOI:** 10.1371/journal.pone.0247399

**Published:** 2021-02-25

**Authors:** Edyta Koman-Wierdak, Katarzyna Nowomiejska, Agnieszka Brzozowska, Dominika Nowakowska, Mario Damiano Toro, Vincenza Bonfiglio, Michele Reibaldi, Teresio Avitabile, Robert Rejdak

**Affiliations:** 1 Department of General Ophthalmology, Medical University of Lublin, Lublin, Poland; 2 Departament of Mathematics and Medical Biostatistics, Medical University of Lublin, Lublin, Poland; 3 Faculty of Medicine, Collegium Medicum Cardinal Stefan Wyszynski University, Warsaw, Poland; 4 Department of Experimental Biomedicine and Clinical Neuroscience, Ophthalmology Section, University of Palermo, Palermo, Italy; 5 Department of Ophthalmology, University of Catania, Catania, Italy; 6 Department of Surgical Sciences, Eye Clinic Section, University of Turin, Turin, Italy; Bascom Palmer Eye Institute, UNITED STATES

## Abstract

The aim of the study is to evaluate the progression of visual field (VF) defects over 16 years of observation and to assess abnormalities in vessels and retinal nerve fibre layer (RNFL) thickness in patients with optic disc drusen (ODD). Both static automated perimetry (SAP) and semi-automated kinetic perimetry (SKP) were performed in 16 eyes of 8 patients (mean age 54 years) with ODD among 26 eyes of 13 patients examined 16 years before. The area of I2e, I4e, III4e, and V4e isopters was measured in deg^2^. The MD and PSD parameters were estimated using SAP. Optical coherence tomography angiography (OCT-A) was additionally performed in 16 ODD eyes and 16 eyes of 8 healthy subjects to estimate the RNFL thickness and vessel density of the optic nerve disc and the macula. The differences in all isopter areas of SKP and SAP parameters after 16 years were not significant. The analysis of OCT-A showed a significant reduction of the vessel density and RNFL of the peripapillary area in each segment in patients with ODD, compared with the control group. The highest reduction of RNFL was observed in the superior segment of the optic disc area (92.56μm vs 126.63μm) also the macular thickness was decreased in ODD patients, compared with the control group. In the macula, there was a significant vascular defect in the whole superficial layer and in the parafoveal deep layer. A strong significant correlation of the parafoveal deep plexus with MD and PSD parameters was detected. In conclusion, VF loss due to ODD after 16 years of the follow-up was not significant both in SKP and SAP. ODD caused a reduced vessel density and RNFL, as well as macular thickness in OCT-A. SAP parameters were influenced by parafoveal deep plexus.

## Introduction

Optic disc drusen (ODD) are acellular deposits located in the optic nerve head of up to 2.4% of the population and are bilateral in approximately 75–91% of cases [1]. Their pathophysiology remains unknown, but they may develop as by‐products of impaired axonal metabolism in genetically predisposed individuals [1, 2]. ODD can cause severe defects of the visual field (VF) and a decrease in the retinal nerve fibre layer (RNFL) and may be accompanied by vessel complications [2]. The prevalence of VF defect varies greatly between studies: from 75% of patients showing no or minimal VF defect [3] to 86% of patients showing VF defects [4]. This inconsistency may be attributed to the difference in the proportion of various types of ODD in each study population, as the type of ODD can influence the presence of VF defect [5]. Asymptomatic ODD are associated with VF defects in up to 87% of cases [1, 2]. These defects are more commonly observed in eyes with visible ODD [5, 6]. Studies suggest that the evolution of ODD involves a transition phase in adolescence, during which VF defects may rapidly progress with minimal worsening thereafter [5].

Long-term evolution of VF defects in ODD patients is important to explore the pathophysiology and prognosis of this condition [6]. In 2009, a perimetric study comparing semi-automated kinetic perimetry (SKP) and static automated perimetry (SAP) was performed on 26 eyes with ODD [7]. The conclusion was that it is necessary to perform both SAP and SKP in patients with ODD due to the diversity of VF defects.

One goal of this prospective study was to assess the progression and compare VF results obtained with SKP and SAP in patients with bilateral visible ODD examined 16 years earlier. Another goal of the study was to measure RNFL and vessel density with OCT-A as these measurements have become currently a more widely used tool for detection of optic nerve pathologies.

## Material and methods

This prospective, single-centre, case-control study was based on the follow‐up examinations of patients with superficial ODD included in the study conducted by Nowomiejska and colleagues published in 2009. Informed consent was obtained from all patients. The study adhered to the tenets of the Declaration of Helsinki. A written approval of the Ethics Committee at the Medical University of Lublin, Poland was given (approval 0254/163/2017).

Attendance in the study performed between 2003 and 2005 in the outpatient clinic at the Department of Ophthalmology in Lublin, Poland, and the ability to take part in the study were the inclusion criteria. Any co‐existing ocular diseases that could potentially affect VF, as glaucoma, anterior ischaemic optic neuropathy, epiretinal membrane, macular hole, age-related macular degeneration, were exclusion criteria. Finally, the analysis included 16 eyes (8 patients) with ODD among 26 eyes (13 patients) that had been examined in the study 16 years ealier. Among absent patients, one patient died, two were unavailable (moved to another city), one patient did not agree to the second examination, and one was diagnosed with glaucoma in both eyes and epiretinal membrane in the left eye. None of them had any eye surgery before. Both eyes of each patient were tested (16 eyes). RNFL, macular thickness and VD were studied in 16 eyes with ODD and compared with 16 healthy eyes of the control group.

Detailed medical history was taken from each patient, both ODD and control group. All patients underwent ophthalmological examination of the best-corrected visual acuity (BCVA) in the Snellen decimal scale, slit‐lamp biomicroscopy, intra‐ocular pressure (IOP) measurement, fundus photography, VF (SAP and SKP) examination, OCT-A (Optovue, Fremont, CA, USA), ultrasound, and fundus autofluorescence (FAF) (Heidelberg Engineering, Germany). The absence of systemic disorders (diabetes mellitus, cardiovascular diseases) and ocular pathology (especially optic nerve disease, macular abnormalities and hyperopia or myopia) and normal results of SAP and SKP were the inclusion criteria for the control group.

To perform SAP, SITA Fast 30‐2 program of Humphrey Field Analyser II (Carl Zeiss Meditec Inc., Dublin, CA, USA) was used in all patients. The same instrument was used currently and 16 years ago. SKP was performed using the Octopus 900 Perimeter (Haag‐Streit Inc., USA), which is a new version of Octopus 101 used for the first examination 16 years ago. SKP was performed with the same three isopters to assess 90° of VF in SKP: III4e and I4e in all cases and, additionally, I2e (in 14 eyes), V4e (2 eyes of the same patient). The angular velocity of the stimulus was kept constant at 3° per second. Appropriate near refraction was provided for each patient inside the central 30° of VF. SAP VF results were classified in the same manner as in the study published in 2009: normal, paracentral scotomas, arcuate defects, and concentric constriction of VF [7].

The vessel density (VD), RNFL of the optic nerve disc and the macular thickness were measured by OCT-A (AngioVue, US)device. The area of the OCT-A scans was 4.5x 4.5mm for the optic disc and 6x6 mm for the macula.

### Statistical analysis

The statistical computations were carried out with STATISTICA 13.0 (StatSoft, Poland) software. The values of the measurable parameters were presented using the mean value, median, and standard deviation (SD), and the non-measurable parameters were expressed as counts and percentages. A two-tailed Mann-Whitney u test was used to compare two independent groups of continuous variables. A Wilcoxon test was used to assess the dependent variables. Spearman’s R correlations were used to determine the relationship between variables. The value of R higher than 0.7 was considered a strong correlation and 0.5 as moderate correlation. A level of significance of p <0.05 was assumed to indicate statistically significant differences or dependencies. The Generalized Estimating Equation (GEE) approach was used to assess factor influencing visual field results. In order to asses predictors for parameters in SAP (MD and PSD), different variables from OCTA examination were used. A statistical model was created, using the maximum likelihood estimator of OCTA parameters for MD and PSD parameters. Significance of the statistical model was confirmed using Wald statistics and probability level.

The sample size was estimated on the basis of the whole capillary parameter mean values obtained in OCT-A and calculation was performed in regard to the healthy controls. Assuming α = 0.05 and the power of the test being 0.95 the minimum sample size was calculated as 14 eyes in each group (Table 1). In order to supplement the statistical analysis, with values additional to p-value, the magnitude of d Cohen (Es) effect was estimated, while calculating the sample size on the basis of the whole capillary parameter of OCT-A in both study and control group.

**Table 1:** Statistical basis for calculation of the sample size of the studied group.

Parameter	Value
Population Mean Mu1	49.5400
Population Mean Mu2	36.4900
Population S.D. (Sigma)	10.0000
Standardized Effect (Es)	1.3050
Type I Error Rate (Alpha)	0.0500
Critical Value of t	2.0555
Power Goal	0.9000
Actual Power for Required N	0.9135
Required N (per group)	14.0000

## Results

All of participants (ODD and control group) were females. The median age of the patients was 54.5 years (Min–Max: 41–72 years). The median BCVA was 0.9 (Min–Max: 0.6–1.0) and the mean IOP was 15 mmHg. The median follow-up time was 16 years (Min–Max: 15–17 years). During the first examination 16 years earlier, the median age was 42 years (Min–Max: 21–76 years) and the median best refracted BCVA was 0.9 (Min–Max: 0.3–1.0). The mean age in the control group was 51.4 years, and the mean BCVA was 1.0 (median 1.0).

### Perimetry

#### Static automated perimetry

The mean MD was -7.44 dB (SD ±6.94dB) during the first examination and -9.54dB (SD ±7.01dB) during the second examination (p = 0.24). The median MD during the first and second examinations was -11.87dB and -16.62 dB, respectively. The mean PSD was 9.09 dB (SD 9.70) during the first examination and 7.55dB (SD 8.48) during the second examination (p = 0.33). The median PSD was 11.17dB and 11.24dB, respectively. The statistical analysis showed no significant differences in the MD and PSD values between the current examination and examination 16 years ago. The classification of VF results obtained in the same ODD patients during the current examination and the examination performed 16 years ago with SKP and SAP is presented in Table 2. The most noticeable changes in VF were arcuate defects (50% in the first vs 37.5% in the second examination).

**Table 2:** Visual field (VF) defects in static automated perimetry in optic disc drusen (ODD) eyes during the first and second examinations.

VF defects	Number of eyes (%) during the 1^st^ examination (n = 16)	Number of eyes (%) during the 2^nd^ examination (n = 16)
**Paracentral scotoma**	2 (12.5%)	3 (18.75%)
**Arcuate defects**	8 (50%)	6 (37.5%)
**Concentric constriction**	3 (18.75%)	4 (25%)
**Normal VF**	3 (18.75%)	3 (18.75%)

n—number of eyes.

#### Semi-automated kinetic perimetry

The mean VF area of all isopters during the first SKP examination was 6017.65 deg², whereas the mean size of VF in SKP in the second examination was 4337.36 deg², which accounted for 27.9% of the area of the initial isopters. The differences in the area of isopters between the first and second examinations were not significant for the isopters separately (V4e, III4e, I4e, and I2e) (Table 3).

**Table 3:** Mean and median area of isopters (deg²) during the first and second examinations with semi-automated kinetic perimetry (SKP) after 16 years of the follow-up in eyes with optic disc drusen (ODD).

Isopters	Number of eyes	mean SKP (deg²) 1^st^	mean SKP (deg²) 2^nd^	median SKP (deg^2^) 1^st^	median SKP (deg^2^) 2^nd^	p- value
**V4e**	2	9631.75	8848.6	10873.3	9383.9	0.18
**III4e**	16	7967.88	6906.76	11920.2	9845.9	0.38
**I4e**	16	5450.66	4116.43	8071.55	6171.55	0.44
**I2e**	14	2349.65	2552.31	3251.7	2357.0	0.22

**Fig 1:**
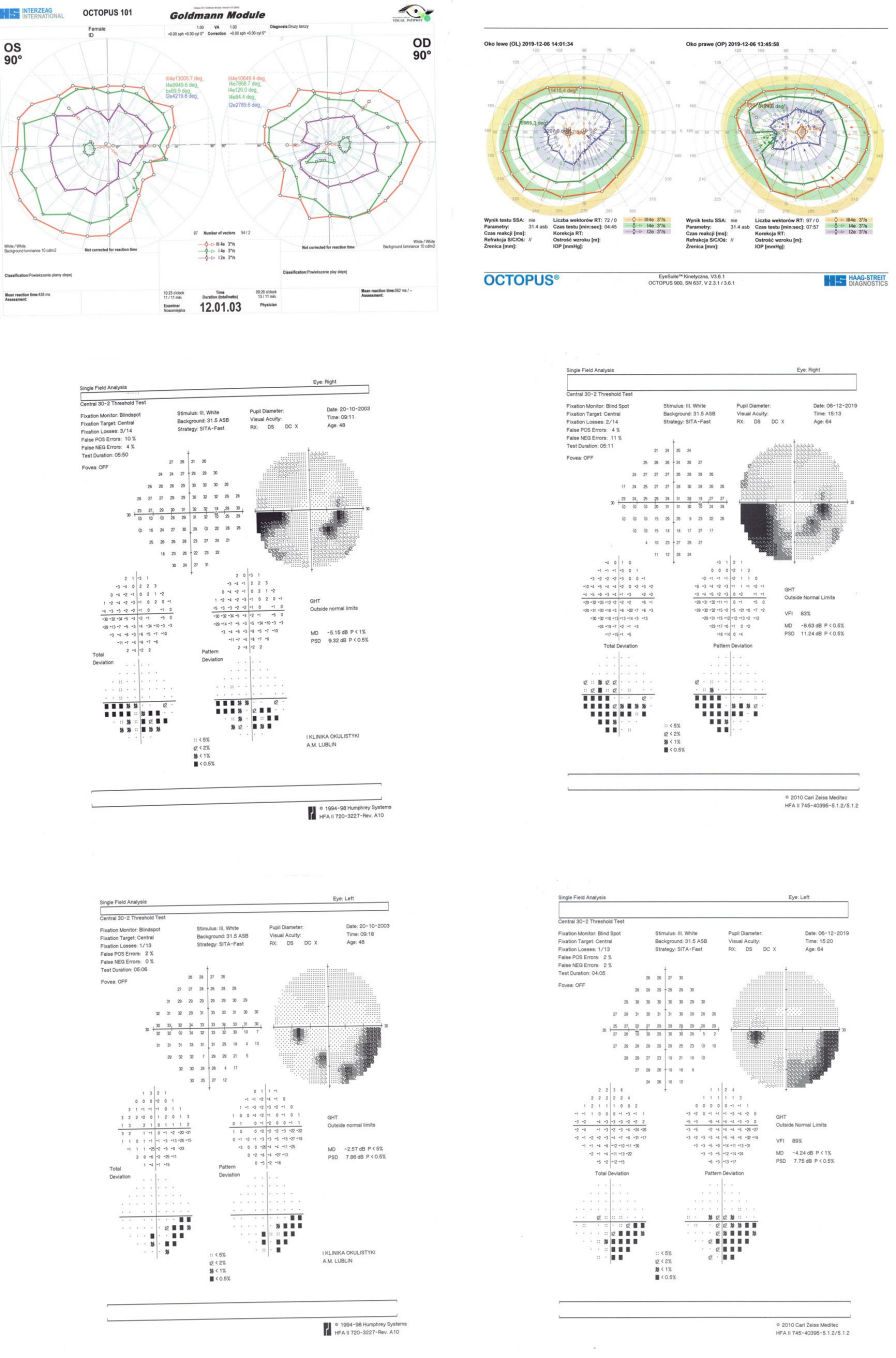
Semi-automated kinetic perimetry (SKP) and static automated perimetry (SAP) performed during the first (left side) and second (right side) examination of a patient with optic disc drusen. A. SKP of both eyes during the first (left side) and second (right side) examinations. Three isopters are used: III4e, I4e, and I2e. B. SAP of the right eye during the first (left side) and second (right side) examinations. C. SAP of the left eye during the first (left side) and second (right side) examinations.

Examples of SKP and SAP results of one of the patients during the first and second examinations are presented below (Fig 1).

### Optical coherence tomography-angiography

We observed a significant reduction of the VD in each segment in the optic nerve head area in eyes with ODD, compared with the control group (Table 4).

**Table 4:** Vessel density (VD) measurements of all vessels or capillaries in the whole image (4.5x4.5mm) and the peripapillary area in eyes with optic disc drusen (ODD) and controls.

Vessel density %	ODD patients			Control group			Statistical analysis	
	Mean	Median	SD	Mean	Median	SD	Z-Mann-Whitney test value	p-value
**Whole capillary**	36.49	35.70	7.07	49.54	49.75	2.75	-4.32	0.00002*
**All vessels**	42.85	42.25	6.91	55.40	55.40	2.32	-4.35	0.00001*
**Peripapillary capillary**	36.14	34.75	9.33	53.19	54.45	3.79	-4.18	0.00003*
**Peripapillary vessels**	42.20	40.50	8.59	57.84	59.10	3.37	-4.22	0.00002*

* statistically significant; SD- standard deviation.

**Fig 2:**
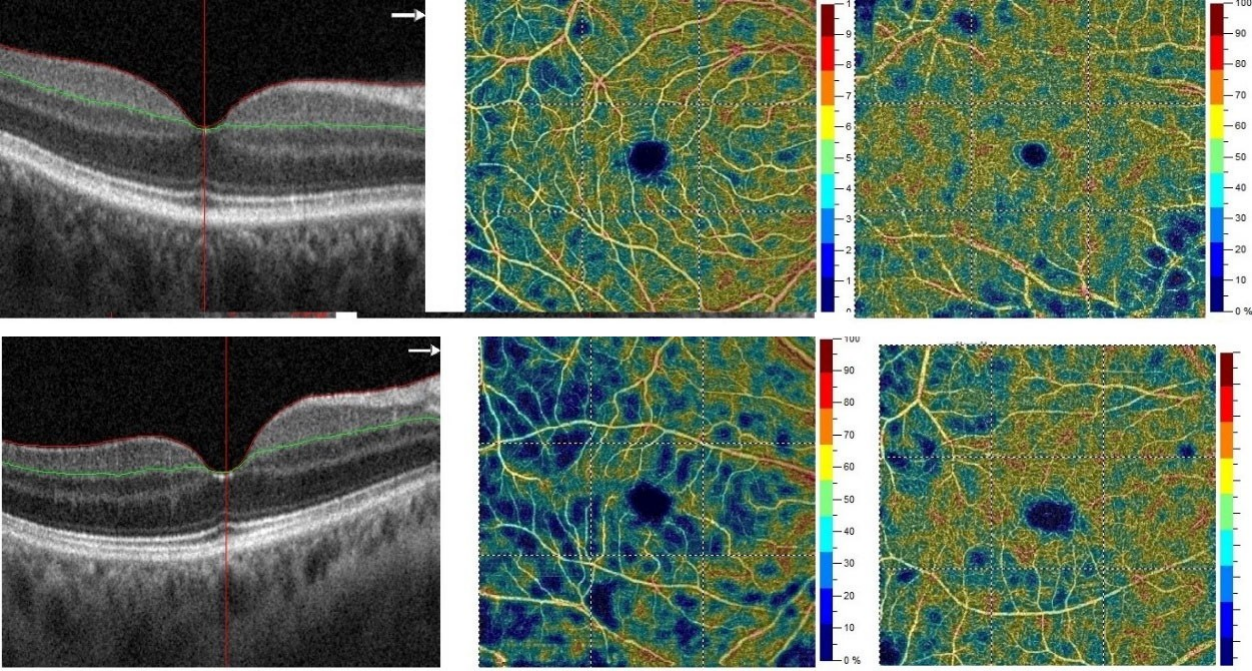
Optical coherence tomography -angiography (OCT-A) of the right eye with optic disc drusen (ODD) and a control group (CG) patient. Respectively: B-scan of the macula, macular superficial capillary plexus layer, and macular deep capillary plexus layer. The non-perfusion areas in the superficial layer in the eye with ODD are larger.

We also analysed VD in the macular region by 6x6 mm OCT-A scans (whole region, fovea, and parafovea) in the superficial and deep layers (Fig 2). Differences between the ODD eyes and the control group were significant only for the whole superficial layer (Table 5).

**Table 5:** Vessel density (VD) in superficial and deep layers in three areas: whole 6x6mm area, fovea, and parafovea in eyes with optic disc drusen (ODD) and in the control group.

Vessel density %	Eyes with ODD			Control group			Statistical analysis	
	Mean	Median	SD	Mean	Median	SD	Z-Mann-Whitney test value	p-value
**Whole superficial**	40.80	41.50	4.89	47.84	47.80	3.51	-3.73	0.0002*
**Whole deep**	46.24	48.15	6.16	49.71	50.30	5.20	-1.39	0.16
**Fovea superficial**	20.78	19.40	9.13	20.31	20.70	6.42	0.00	1.00
**Fovea deep**	36.43	37.20	12.72	36.82	37.15	5.49	0.08	0.94
**Parafovea sup.**	45.34	47.00	6.16	48.78	49.75	5.38	-1.90	0.06
**Parafovea deep**	51.21	52.35	4.45	54.50	55.15	3.74	-2.02	0.04*

*: statistically significant; SD- standard deviation.

**Fig 3:**
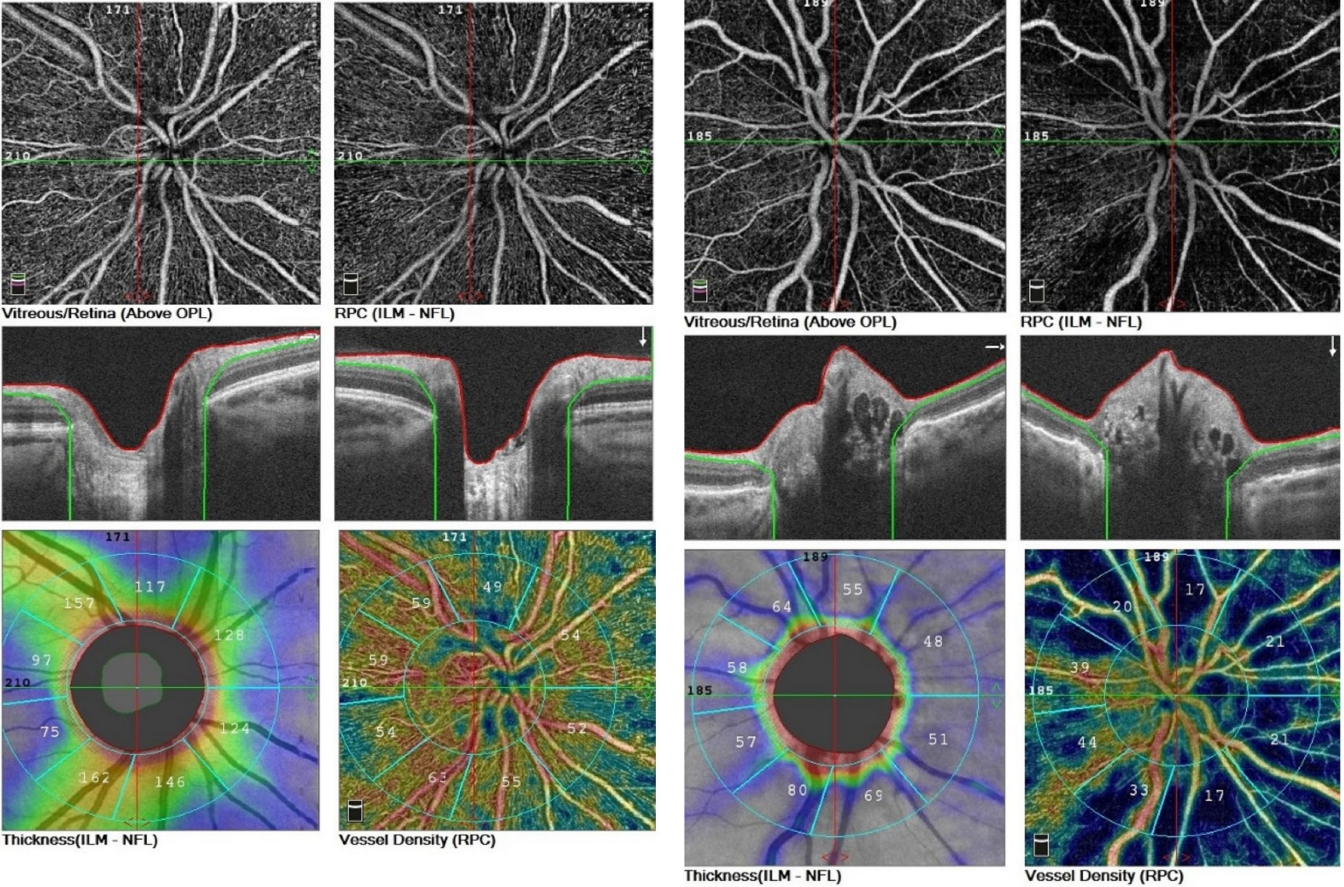
The optic disc OCT-A of the right eyes, in eye with optic disc drusen (the left side) and the control group (the right side). In sequence: nonperfusion areas in the radial peripapillary capillaries; B-scans of optic nerve head; coloured coded density maps; reduced RNFL and vascular density consistent with the blue areas.

The analysis showed a significant reduction of the peripapillary RNFL in the eyes with ODD compared with the control group (Table 6). Considering each sector separately, the superior sector showed the highest reduction of RNFL compared with the control group. The RNFL thickness decrease and increase of non-perfusion area in the radiant peripapillary capillary is observed in the ODD eye (Fig 3).

**Table 6:** Retinal nerve fibre layer (RNFL) thickness in μm in the optic disc area of 4.5x4.5mm and in each sector in eyes with optic disc drusen (ODD) and the control group.

Peripapillary RNFL thickness (μm)	Eyes with ODD			Control group			Statistical analysis	
	Mean	Median	SD	Mean	Median	SD	Z-Mann-Whitney test value	p-value
**Overall**	85.38	72.000	26.95	111.06	111.50	10.08	-2.45	0.01*
**Superior**	92.56	82.000	34.54	126.63	135.50	28.20	-2.79	0.01*
**Nasal**	73.63	70.000	24.96	101.31	99.50	12.69	-3.09	0.002*
**Inferior**	101.13	95.000	36.63	135.56	136.50	15.61	-3.13	0.002*
**Temporal**	73.50	73.500	17.71	75.19	77.50	10.15	-0.28	0.78

*: statistically significant; SD- standard deviation.

The analysis of macular thickness showed a significant decrease in each measured area within 6x6mm, except the parafoveal region, in eyes with ODD (Table 7).

**Table 7:** Macular thickness in μm in the macular region of 6x6mm in eyes with optic disc drusen (ODD) and the control group.

Macular thickness (μm)	ODD patients			Control group			Statistical analysis	
	Mean	Median	SD	Mean	Median	SD	Z-Mann-Whitney test value	p-value
**Whole**	272.69	276.50	19.69	304.00	299.00	12.92	-4.37	0.00001*
**Superior hemisphere**	276.13	280.00	19.37	304.63	302.00	11.74	-4.45	0.00001*
**Inferior hemisphere**	271.00	277.00	21.46	301.13	298.00	13.65	-4.05	0.0001*
**Parafovea**	311.31	319.00	33.91	330.81	330.00	10.45	-1.73	0.083

* statistically significant; SD- standard deviation.

There was no significant correlation between the VD measurements of all vessels or capillaries in the whole image (4.5x4.5mm) and the peripapillary area and between the isopter area and SAP parameters.

There was a moderate correlation only between the temporal peripapillary RNFL and the III4e isopter (R = 0.54; p = 0.03) and MD (R = -0.64; p = 0.05).

A strong significant correlation of the parafoveal deep plexus with MD (R = 0.73; p = 0.02), and PSD (R = 0.7; p = 0.02) parameters was also detected, as well as moderate correlation with I4e isopter (R = 0.61; p = 0.01),

The correlations of the macular thickness with the area of isopters and SAP parameters were moderate (R value between 0.5 and 0.7) and are presented in Table 8.

**Table 8:** Correlations of the macular thickness with the area of three isopters and SAP parameters (MD and PSD).

OCT-A parameters for macular thickness	I4e		III4e		I2e		MD		PSD	
	R	p	R	p	R	p	R	p	R	p
**Whole**	-0.32	0.22	0.54	0.03	-0.58	-0.58	-0.67	0.03	-0.43	0.21
**Superior hemi**	-0.12	0.66	0.62	0.01	-0.48	-0.48	-0.59	0.07	-0.31	0.38
**Inferior hemi**	-0.19	0.48	0.56	0.02	-0.47	-0.47	-0.69	0.03	-0.44	0.21
**Parafovea**	-0.24	0.37	0.49	0.05	-0.48	-0.48	-0.56	0.09	-0.28	0.43

R—Spearman’s rank correlation; p-value.

The maximum likelihood estimator was 3.58 for MD and 2.901 for PSD.

The statistical model using the maximum likelihood estimator of OCTA parameters for MD was as follows: D = 0.21· Parafovea deep (p<0,000001)-0,03·Temporal (p<0,000001) -0,08·Whole sup. (p = 0,0008)-3.58.

The statistical model using the maximum likelihood estimator of OCTA parameters for MD was as follows: PSD = 0.042· Whole deep (p<0,000001) -0,026·Superior (p<0,000001)+0,061·Inferior (p<0,000001)-2.901.

Other parameters of OCT-A for SAP as well as all OCT-A parameters for isopters’ area of SKP were not significant in this model.

## Discussion

This is the first prospective study to follow-up VF in ODD patients with the use of SAP and SKP with a median follow‐up period of 16 years. Additionally, OCT-A of the optic nerve and the macula was performed during the last examination to determine the structure-function relationship.

Our study shows a decrease in the area of all isopters obtained with SKP over the period of 16 years, but it was not statistically significant. We also did not observe any significant progression of the retinal sensitivity loss in SAP. This could be explained by the limited time frame and also the small sample size of the patients that were studied. However, several studies have shown a correlation between age and VF deterioration in ODD patients, suggesting a slowly progressive VF loss [3, 8].

In the first study published in 2009 [7], we demonstrated the diversity of VF defects in ODD. The most common changes during the first and second examinations were arcuate defects. It is known that VF defects are more severe with visible ODD and those with thinning of the peripapillary RNFL in OCT imaging [9]. Up to 87% of adults have VF abnormalities and those with visible drusen have more VF defects [6, 10]. Central visual acuity is preserved in most cases of ODD unless there is secondary pathology (macular pathology or ischaemic optic injury) [3]. It is well known that retinal sensitivity decreases with age in healthy eyes. Zulauf and co-workers [11] observed a significant linear decrease in retinal sensitivity with increasing age and estimated the mean sensitivity index at 0.064 dB/year of life.

Lee and Zimmerman [3] performed a 36-year follow-up study using Goldmann manual kinetic perimetry and observed a 1.6% rate of VF loss in 32 ODD patients. In a longitudinal study (56 years of follow-up), Malmqvist et al. [8] described a 27% decrease in the Goldmann perimetry area in ODD eyes. We observed loss of 27.9% of the area of all isopters in SKP during the 16-year follow-up.

In our study, with the mean follow-up period was 16 years and the median age of patients 42 years, we did not observe any significant progression of the VF defects in eyes with ODD, both with SAP and SKP. Frisen [12] showed severe progression in the size and number of ODD in a patient from the age of 8–16. At the age of 31, there were no changes in ODD appearance, compared to a fundus photo taken 16 years before. It is possible that the transition phase wherein buried ODD progresses to superficial ODD represents a period with most rapidly progressive VF defects [8]. The slow progression in VF defects in adult patients may be explained by the stabilized ODD anatomy, as the peripheral parts of VF are represented by a lower number of ganglion cells than in the central part [8, 13].

OCT-A provides non-invasive and reliable data which allows measurements of microvascular changes and RNFL defects and comparison of results with a control group. Our patients showed a decrease in the vascular density on the optic nerve head and in the macular region in OCT-A. OCT-A imaging of optic disc revealed reduced vascular density in all ODD eyes, especially in the peripapillary capillary area of the optic disc. Gaier and colleagues [14] detected microvascular attenuation in OCT-A consistent with VF defect in a patient with ODD. The authors also reported macular microvascular defects only in the superficial capillary plexus. Another OCT-A study showed that ODD patients had a lower flow index and reduced vascular density on the optic nerve head compared to the control group [15]. Macular OCT-A revealed a decrease in vascular density, suggesting ischaemia in the superficial and deep capillary plexus layers bilaterally [15, 16]. Decrease in macular vascular density was found also in our group of patients with ODD, particularly in the superficial capillary plexus layer. This may be related to the superficial location of ODD, which appear to cause more compression of the upper capillary layer [14, 16]. Therefore, areas of reduced vascular density detected by OCT-A may be a predictor of future paracentral scotoma [17].

In our work, the RNFL thickness was significantly reduced in the whole optic disc (the most pronounced reduction in the superior sector) in the ODD patients in comparison with the control group. Authors of several studies have shown a relationship between RNFL thinning and perimetric parameters in patients with ODD [3, 5, 18, 19]. Malmqvist and colleagues [5] showed a positive correlation between the mean deviation and the RNFL thinning in a group of 149 ODD eyes. Engelke et al. [20] described a positive correlation between RNFL and vessel density, suggesting an association of microvascular narrowing with RNFL thinning. This was supported by data on the ganglion cell complex (GCC), which was decreased in the ODD group and showed a significant positive correlation with the VD [20, 21].

Several studies have found a decreased global peripapillary RNFL thickness in patients with superficial ODD compared to patients with buried ODD [18, 22]. In a study by Sato el al. [23], a significant negative correlation between ODD and RNFL thickness in the optic disc area was found. Localized peripapillary RNFL thinning corresponding to the quadrant with the highest aggregation of ODD was reported by Roh et al. [18]. Another study reported a macular RNFL decrease in patients with ODD compared to control subjects [17]. Both peripapillary RNFL thickness and macular thickness are needed to assess their relation to sectoral crowding and VF defects [1]. In the present study we found that selected parameters of macular thickness in OCT-A may significantly affect SAP parameters. Parafovea deep, temporal and whole superior parameters of OCT-A influenced the MD of SAP. Whole deep, superior and inferior influenced the PSD parameter of SAP. In a recent study conducted by Yan [24], the parameters of OCT-A, which correlate well with VF loss in SAP, were investigated. Special custom quantification software with an interactive interface was created for this purpose. The parameters were as follows: peripapillary RNFL, macular ganglion cell complex (GCC), peripapillary vessel area density (VAD), macular vessel diameter (VD), and flux.

In this study, we have observed a dramatic decrease in the vessel density and retinal nerve fibre layer thickness in eyes of patients with ODD compared to normal. It is surprising that this did not result in a more dramatic loss of visual field. However, it was not possible to compare the OCTA values at the baseline examination, as this device was not available 16 years before. Thus, we can suspect that the ischaemic injury due to the pressure of ODD was present also at the beginning of the follow-up.

We are aware that there are some limitations of the present study: the limited number of patients, different devices used for SKP examinations, and the use of longitudinal information from visual field testing from only two points in time (current and 16 years before).

In conclusion, VF loss due to ODD was not significant after 16 years of the follow-up, as revealed by both SKP and SAP. The analysis of OCT-A showed a significant reduction of the vessel density and RNFL of the peripapillary area in each segment in patients with ODD, compared with normal eyes. Correlations among VF, RNFL loss, and microvascular changes determined in a larger group of patients and follow-up studies would help to characterize the relationships of microvascular alterations with nerve fibre layer loss in the presence of ODD.

In our opinion, patients with ODD should regularly undergo ophthalmic examinations focused on VF testing, RNFL, and vessel density analysis. The OCT-A evaluation of the optic nerve head and the macula may play an important role in early detection of ischaemic complications and RNFL loss in ODD patients.

## Supporting information

S1 File (DOCX)

S2 File (DOCX)

S3 File (XLSX)

## References

[1] Hamman S, Malmqvist L, Costello F.Optic disc drusen: understanding an old problem from a new perspective. Acta Ophthalmol. 2018;96: 673–684. 10.1111/aos.13748 29659172

[2] Lorentzen SE. Drusen of the optic disk. A clinical and genetic study. Acta Ophthalmol (Copenh) 1966; 90(Suppl): 1–180. 6012937

[3] Lee AG, Zimmerman MB. The rate of visual field loss in optic nerve head drusen. Am J Ophthalmol. 2005;139: 1062–1066. 10.1016/j.ajo.2005.01.020 15953437

[4] Mustonen E. Pseudopapilloedema with and without verified optic disc drusen. A clinical analysis II: visual fields. Acta Ophthalmol (Copenh). 1983;61(6):1057–66. 6659908 10.1111/j.1755-3768.1983.tb01493.x

[5] Malmqvist L, Wegener M, Sander BA, Hamann S. Peripapillary retinal nerve fiber layer thickness corresponds to drusen location and extent of visual field defects in superficial and buried optic disc drusen. J Neuroophthalmol. 2016;36: 41–45. 10.1097/WNO.0000000000000325 26720518

[6] Wilkins JM, Pomeranz HD. Visual manifestations of visible and buried optic disc drusen. J Neuroophthalmol. 2004;24(2):125–129. 10.1097/00041327-200406000-00006 15179065

[7] Nowomiejska K, Rejdak R, Zagórski Z & Żarnowski T. Comparison of static automated perimetry and semi‐automated kinetic perimetry in patients with bilateral visible optic nerve head drusen. Acta Ophthalmol. 2009;87(7): 801–805. 10.1111/j.1755-3768.2008.01364.x 18721249

[8] Malmqvist L, Lund‐Andersen H, Hamann S. Long‐term evolution of superficial optic disc drusen. Acta Ophthalmol. 2017;95: 352–356. 10.1111/aos.13315 27996202

[9] Noval S, Visa J, Contreras I. visual field defects due to optic disk drusen in children. Graefes Arch Clin Exp Ophthalmol. 2013;251(10):2445–2450. 10.1007/s00417-013-2384-6 23733034

[10] Flores-Rodriguez P, Gili P, Martin-Rios MD. Ophthalmic features of optic disc drusen. Ophthalmologica. 2012;228(1):59–66. 10.1159/000337842 22584542

[11] Zulauf M, LeBlanc RP, Flammer J. Normal visual fields measured with Octopus‐Program G1. II. Global visual field indices. Graefes Arch Clin Exp Ophthalmol. 1994;232: 516–522. 10.1007/BF00181993 7959089

[12] Frisen L. Evolution of drusen of the optic nerve head over 23 years. Acta Ophthalmol. 2008;86: 111–112. 10.1111/j.1600-0420.2007.00986.x 18233998

[13] Katz BJ, Pomeranz HD. Visual field defects and retinal nerve fiber layer defects in eyes with buried optic nerve drusen. Am J Ophthalmol. 2006;141: 248–253. 10.1016/j.ajo.2005.09.029 16458676

[14] Gaier ED, Rizzo JF 3rd, Miller JB, Cestari DM. Focal Capillary Dropout Associated With Optic Disc Drusen Using Optical Coherence Tomographic Angiography. J Neuroophthalmol. 2017;37(4):405–410. 10.1097/WNO.0000000000000502 28520583

[15] Cennamo G, Tebaldi S, Amoroso F, Arvanitis D, Breve M, Cennamo G. Optical Coherence Tomography Angiography in Optic Nerve Drusen. Ophthalmic Res. 2018; 59:76–80. 10.1159/000481889 29186723

[16] Flores-Reyes E, Hoskens K, Mansouri K. Optic Nerve Head Drusen: Imaging Using Optical Coherence Tomography Angiography. J Glaucoma. 2017;26:845–849. 10.1097/IJG.0000000000000730 28767460

[17] Biçer O, Atilla H. Microvascular Changes Associated with Optic Disc Drusen: Case Report. Turk J Ophthalmol. 2019;49(5): 300–304. 10.4274/tjo.galenos.2019.14194 31650815 PMC6823588

[18] Roh S, Noecker RJ, Schuman JS, Hedges TR 3rd, Weiter JJ, Mattox C. Effect of optic nerve head drusen on nerve fiber layer thickness. Ophthalmology. 1998;105: 878–885. 10.1016/S0161-6420(98)95031-X 9593392 PMC1937403

[19] Gili P, Flores‐Rodriguez P, Martin‐Rios MD, Carrasco Font C. Anatomical and functional impairment of the nerve fiber layer in patients with optic nerve head drusen. Graefes Arch Clin Exp Ophthalmol. 2013;251: 2421–2428. 10.1007/s00417-013-2438-9 23955723

[20] Engelke H, Shajari M, Riedel J, Mohr N, Priglinger SG, Mackert MJ. OCT angiography in optic disc drusen: comparison with structural and functional parameters. Br J Ophthalmol. 2019; 0:1–5. 10.1136/bjophthalmol-2019-314096 31744797

[21] Casado A, Rebolleda G, Guerrero L. Measurement of retinal nerve fiber layer and macular ganglion cell-inner plexiform layer with spectral-domain optical coherence tomography in patients with optic nerve head drusen. Graefes Arch Clin Exp Ophthalmol. 2014;252:1653–60. 10.1007/s00417-014-2773-5 25128962

[22] Mistlberger A, Sitte S, Hommer A, Emesz M, Dengg S, Hitzl W et al. Scanning laser polarimetry (SLP) for optic nerve head drusen. Int Ophthalmol. 2001;23: 233–237. 10.1023/a:1014401202762 11944846

[23] Sato T, Mrejen S, Spaide RF. Multimodal imaging of optic disc drusen. Am J Ophthalmol. 2013;156: 275–282. 10.1016/j.ajo.2013.03.039 23677136

[24] Yan Y, Zhou X, Chu Z, Stell L, Shariati MA, Wang RK et al. Vision loss in optic disc drusen correlates with increased macular vessel diameter and flux and reduced peripapillary vascular density. Am J Ophthalmol. 2020;28:S0002-9394(20)30187-2. 10.1016/j.ajo.2020.04.019 32360344 PMC8186883

