# Supplementary material for: Kinetic and static perimetry after 16 years and additional OCT-A analysis in eyes with long-lasting optic disc drusen
Source: PLoS One. 2021 Feb 25;16(2):e0247399. doi: 10.1371/journal.pone.0247399 (PMC7906410; doi:10.1371/journal.pone.0247399)
Supplement: S1 File — (DOCX) [file pone.0247399.s001.docx]

**Statistical analysis**

**Table 1. Vessel density (VD) measurements of all vessels or capillaries in the whole image (4.5x4.5mm) and the peripapillary area in eyes with optic disc drusen (ODD) and controls**

| **Parameters** | **ODD group** | | | **Control group** | | | **Statistical analysis** | |
| --- | --- | --- | --- | --- | --- | --- | --- | --- |
|  | **Maea** | **Median** | **SD** | **Maen** | **Median** | **SD** | **Z** | **p** |
| Whole capillary | 36,49 | 35,70 | 7,07 | 49,54 | 49,75 | 2,75 | -4,32 | 0,00002* |
| All capillary | 42,85 | 42,25 | 6,91 | 55,40 | 55,40 | 2,32 | -4,35 | 0,00001* |
| Peri. capillary | 36,14 | 34,75 | 9,33 | 53,19 | 54,45 | 3,79 | -4,18 | 0,00003* |
| Peri. all | 42,20 | 40,50 | 8,59 | 57,84 | 59,10 | 3,37 | -4,22 | 0,00002* |

**Table 2 Retinal nerve fibre layer (RNFL) thickness in µm in the optic disc area of 4.5x4.5mm and in each sector in eyes with optic disc drusen (ODD) and the control group**

| **Parameters** | **ODD** | | | **Control Group** | | | **Statistical analysis** | |
| --- | --- | --- | --- | --- | --- | --- | --- | --- |
|  | **Mean** | **Median** | **SD** | **Mean** | **Median** | **SD** | **Z** | **p** |
| Mean | 85,38 | 72,000 | 26,95 | 111,06 | 111,50 | 10,08 | -2,45 | 0,01* |
| Superior | 92,56 | 82,000 | 34,54 | 126,63 | 135,50 | 28,20 | -2,79 | 0,01* |
| Nasal | 73,63 | 70,000 | 24,96 | 101,31 | 99,50 | 12,69 | -3,09 | 0,002* |
| Inferior | 101,13 | 95,000 | 36,63 | 135,56 | 136,50 | 15,61 | -3,13 | 0,002* |
| Temporal | 73,50 | 73,500 | 17,71 | 75,19 | 77,50 | 10,15 | -0,28 | 0,78 |

**Table 3. Vessel density in superficial and deep layers in three areas: whole 6x6mm area, fovea, and parafovea in eyes with optic disc drusen (ODD) and in the control group**

| **Parameters** | **ODD** | | | **Control group** | | | **Statistical analysis** | |
| --- | --- | --- | --- | --- | --- | --- | --- | --- |
|  | **Mean** | **Median** | **SD** | **Mean** | **Median** | **SD** | **Z** | **p** |
| Whole sup. | 40,80 | 41,50 | 4,89 | 47,84 | 47,80 | 3,51 | -3,73 | 0,0002* |
| Whole deep | 46,24 | 48,15 | 6,16 | 49,71 | 50,30 | 5,20 | -1,39 | 0,16 |
| Fovea sup. | 20,78 | 19,40 | 9,13 | 20,31 | 20,70 | 6,42 | 0,00 | 1,00 |
| Fovea deep | 36,43 | 37,20 | 12,72 | 36,82 | 37,15 | 5,49 | 0,08 | 0,94 |
| Parafovea sup. | 45,34 | 47,00 | 6,16 | 48,78 | 49,75 | 5,38 | -1,90 | 0,06 |
| Parafovea deep | 51,21 | 52,35 | 4,45 | 54,50 | 55,15 | 3,74 | -2,02 | 0,04* |

**Table 4. Retinal nerve fibre layer (RNFL) thickness in µm in the macular region of 6x6mm in eyes with optic disc drusen (ODD) and the control group**

| **Parameters** | **ODD** | | | **Control group** | | | **Statistical analysis** | |
| --- | --- | --- | --- | --- | --- | --- | --- | --- |
|  | **Mean** | **Median** | **SD** | **Maean** | **Median** | **SD** | **Z** | **p** |
| Whole | 272,69 | 276,50 | 19,69 | 304,00 | 299,00 | 12,92 | -4,37 | 0,00001* |
| Superior hemi | 276,13 | 280,00 | 19,37 | 304,63 | 302,00 | 11,74 | -4,45 | 0,00001* |
| Inferior hemi | 271,00 | 277,00 | 21,46 | 301,13 | 298,00 | 13,65 | -4,05 | 0,0001* |
| Parafovea | 311,31 | 319,00 | 33,91 | 330,81 | 330,00 | 10,45 | -1,73 | 0,083 |

Table 5. I4e isopter in 1^st^ and 2nd exam.

| **Group** | **Mean** | **SD** | **Lower quartile** | **Median** | **Upper quartile** |
| --- | --- | --- | --- | --- | --- |
| 1st exam. | 5450,66 | 4934,70 | 1749,75 | 8071,55 | 4065,28 |
| 2nd exam. | 4116,43 | 5093,50 | 1591,65 | 6171,55 | 2411,71 |
| Z=0,78; p=0,44 | | | | | |

Tabela 6. III4e isopter in 1st and 2nd exam.

| **Group** | **Mean** | **SD** | **Lower quartile** | **Median** | **Upper quartile** |
| --- | --- | --- | --- | --- | --- |
| 1st exam. | 7967,88 | 8101,15 | 5520,00 | 11920,2 | 4321,11 |
| 2nd exam. | 6906,76 | 7605,20 | 4298,20 | 9845,9 | 3428,80 |
| Z=0,88; p=0,38 | | | | | |

Table 7. I2e isopter in 1st and 2nd exam

| **Group** | **Mean** | **SD** | **Lower quartile** | **Median** | **Upper quartile** |
| --- | --- | --- | --- | --- | --- |
| 1st exam. | 2349,65 | 2656,80 | 531,100 | 3251,70 | 1905,62 |
| 2nd exam. | 2552,31 | 1811,45 | 902,700 | 2357,00 | 3576,56 |
| Z=1,22; p=0,22 | | | | | |

Table 8. V4e isopter in 1st and 2nd exam.

| **Group** | **Mean** | **SD** | **Lower quartile** | **SD** | **Upper quartile** |
| --- | --- | --- | --- | --- | --- |
| 1st exam. | 9631,75 | 9631,75 | 8390,20 | 10873,30 | 1755,82 |
| 2nd exam. | 8848,60 | 8848,60 | 8313,30 | 9383,90 | 757,03 |
| Z=1,34; p=0,18 | | | | | |

Table 9. MD in 1st and 2nd exam.

| **Group** | **Mean** | **SD** | **Lower quartile** | **Median** | **Upper quartile** |
| --- | --- | --- | --- | --- | --- |
| 1st exam. | 7,44 | 6,94 | 2,57 | 11,87 | 4,96 |
| 2nd exam. | 9,54 | 7,01 | 2,96 | 16,62 | 8,29 |
| Z=1,17; p=0,24 | | | | | |

Table 10. PSD in 1st and 2nd exam.

| **Group** | **Mean** | **SD** | **Lower quartile** | **Median** | **Upper quartile** |
| --- | --- | --- | --- | --- | --- |
| 1st exam. | 9,09 | 9,70 | 7,86 | 11,17 | 3,24 |
| 2nd exam. | 7,55 | 8,48 | 2,99 | 11,24 | 4,24 |
| Z=0,97; p=0,33 | | | | | |

Table 11. Correlations between vessels density (disc) parameters and isopters results

| **Parameters** | **I4e** | | **III4e** | | **I2e** | | **V4e** | | **MD** | | **PSD** | |
| --- | --- | --- | --- | --- | --- | --- | --- | --- | --- | --- | --- | --- |
|  | **R** | **p** | **R** | **p** | **R** | **p** | **R** | **p** | **R** | **p** | **R** | **p** |
| Whole capillary | 0,01 | 0,96 | 0,30 | 0,25 | -0,30 | -0,30 | - | - | -0,41 | 0,24 | -0,31 | 0,38 |
| All capillary | 0,15 | 0,57 | 0,31 | 0,25 | -0,17 | -0,17 | - | - | -0,36 | 0,31 | -0,24 | 0,51 |
| Peri. capillary | -0,34 | 0,23 | 0,52 | 0,06 | -0,05 | -0,05 | - | - | -0,19 | 0,65 | 0,26 | 0,53 |
| Peri. all | -0,32 | 0,26 | 0,48 | 0,08 | -0,02 | -0,02 | - | - | -0,10 | 0,82 | 0,36 | 0,39 |

Table 12. Correlations between disc RNFL and isopters results

| **Parameters** | **I4e** | | **III4e** | | **I2e** | | **V4e** | | **MD** | | **PSD** | |
| --- | --- | --- | --- | --- | --- | --- | --- | --- | --- | --- | --- | --- |
|  | **R** | **p** | **R** | **p** | **R** | **p** | **R** | **p** | **R** | **p** | **R** | **p** |
| Mean | 0,25 | 0,35 | 0,40 | 0,12 | 0,06 | 0,84 | - | - | -0,53 | 0,12 | -0,28 | 0,43 |
| Superior | 0,20 | 0,47 | 0,39 | 0,14 | 0,08 | 0,78 | - | - | -0,61 | 0,06 | -0,38 | 0,27 |
| Nasal | 0,19 | 0,47 | 0,44 | 0,09 | -0,10 | 0,74 | - | - | -0,49 | 0,15 | -0,25 | 0,49 |
| Inferior | 0,08 | 0,77 | 0,42 | 0,10 | 0,04 | 0,89 | - | - | -0,58 | 0,08 | -0,44 | 0,21 |
| Temporal | 0,19 | 0,48 | 0,54 | 0,03 | -0,30 | 0,31 | - | - | -0,64 | 0,05 | -0,52 | 0,13 |

Table 13. Correlations between macular vessels density and isopters results

| **Parameters** | **I4e** | | **III4e** | | **I2e** | | **V4e** | | **MD** | | **PSD** | |
| --- | --- | --- | --- | --- | --- | --- | --- | --- | --- | --- | --- | --- |
|  | **R** | **p** | **R** | **p** | **R** | **p** | **R** | **p** | **R** | **p** | **R** | **p** |
| Whole sup. | -0,37 | 0,16 | 0,08 | 0,77 | 0,45 | 0,10 | - | - | -0,16 | 0,65 | 0,18 | 0,63 |
| Whole deep | 0,35 | 0,19 | -0,06 | 0,81 | 0,41 | 0,15 | - | - | 0,43 | 0,21 | 0,49 | 0,15 |
| Fovea sup. | -0,19 | 0,48 | -0,20 | 0,46 | 0,21 | 0,46 | - | - | 0,03 | 0,93 | 0,42 | 0,23 |
| Fovea deep | -0,04 | 0,89 | -0,24 | 0,36 | 0,43 | 0,13 | - | - | 0,24 | 0,51 | 0,61 | 0,06 |
| Parafovea sup. | 0,26 | 0,33 | -0,16 | 0,56 | 0,32 | 0,26 | - | - | 0,50 | 0,14 | 0,53 | 0,12 |
| Parafovea deep | 0,61 | 0,01 | -0,04 | 0,87 | 0,27 | 0,35 | - | - | 0,73 | 0,02 | 0,70 | 0,02 |

Table 14. Correlations between macular RNFL and isopters results

| **Parametry** | **I4e** | | **III4e** | | **I2e** | | **V4e** | | **MD** | | **PSD** | |
| --- | --- | --- | --- | --- | --- | --- | --- | --- | --- | --- | --- | --- |
|  | **R** | **p** | **R** | **p** | **R** | **p** | **R** | **p** | **R** | **p** | **R** | **p** |
| Whole | -0,32 | 0,22 | 0,54 | 0,03 | -0,58 | -0,58 | - | - | -0,67 | 0,03 | -0,43 | 0,21 |
| Superior hemi | -0,12 | 0,66 | 0,62 | 0,01 | -0,48 | -0,48 | - | - | -0,59 | 0,07 | -0,31 | 0,38 |
| Inferior hemi | -0,19 | 0,48 | 0,56 | 0,02 | -0,47 | -0,47 | - | - | -0,69 | 0,03 | -0,44 | 0,21 |
| Parafovea | -0,24 | 0,37 | 0,49 | 0,05 | -0,48 | -0,48 | - | - | -0,56 | 0,09 | -0,28 | 0,43 |

Table 15. The assesment of differences between isopters in 1st and 2nd exam.

| **Izopters** | **1st exam.** | | | **2nd exam.** | | |
| --- | --- | --- | --- | --- | --- | --- |
|  | **Mean** | **Median** | **SD** | **Mean** | **Median** | **SD** |
| I4e | 5450,66 | 4934,70 | 4065,28 | 4116,43 | 5093,50 | 2411,71 |
| III 4e | 7967,88 | 8101,15 | 4321,11 | 6906,76 | 7605,20 | 3428,80 |
| I2e | 2349,65 | 2656,80 | 1905,62 | 2552,31 | 1811,45 | 3576,56 |
| V4e | 9631,75 | 9631,75 | 1755,82 | 8848,60 | 8848,60 | 757,03 |
| **Statistical analysis** | H=15,69; p=0,001*  III4e i I2e Z=3,67; p=0,001* | | | H=16,00; p=0,001*  III4e i I2e Z=3,68; p=0,001* | | |
