# Supplementary material for: Kinetic and static perimetry after 16 years and additional OCT-A analysis in eyes with long-lasting optic disc drusen
Source: PLoS One. 2021 Feb 25;16(2):e0247399. doi: 10.1371/journal.pone.0247399 (PMC7906410; doi:10.1371/journal.pone.0247399)
Supplement: S2 File — (DOCX) [file pone.0247399.s002.docx]

Statistical analysis 2

Taking into account the mean values ​​for the Whole capillary variable in the study and control group, the test power was estimated to be 0.95.

|  | Value |
| --- | --- |
| Population mean Mi1 | 49.5400 |
| Population mean Mi2 | 36.4900 |
| SD in population (Sigma) | 10.0000 |
| Standardized effect (Es) | 1.3050 |
| Number of group N1 | 16.0000 |
| Number of group N2 | 16.0000 |
| Probability of error (Alfa) | 0.0500 |
| Critical value t | 2.0423 |
| Power | 0.9464 |

Based on the obtained mean results in the groups for the Whole capillary, the size in the groups was calculated, which would allow to demonstrate the statistical significance between the groups. With the Whole capillary results for the control group 49.54 and the ODD group 36.49, assuming α = 0.05 and the test power equal to 0.90, 14 eyes are needed in each group.

|  | Value |
| --- | --- |
| Population mean Mi1 | 49.5400 |
| Population mean Mi2 | 36.4900 |
| SD in population (Sigma) | 10.0000 |
| Standardised effect (Es) | 1.3050 |
| (Alfa) | 0.0500 |
| Critical value t | 2.0555 |
| Power | 0.9000 |
| Power for the sample N | 0.9135 |
| Required N (in group) | 14.0000 |

Assuming the difference between the groups in the Whole capillary 10 values, assuming α = 0.05 and the test power equal to 0.90, 23 eyes are needed in each group.

|  | Wartość |
| --- | --- |
| Mi1 | 50.0000 |
| Mi2 | 40.0000 |
| Sigma | 10.0000 |
| Es | 1.0000 |
| Alfa | 0.0500 |
| t | 2.0154 |
| Power | 0.9000 |
| Power for the sample N | 0.9125 |
| Required N (in group) | 23.0000 |

Individual parameters of OCT related to vessel density were taken into account for the assessment of the MD and PSD predictors. In the Generalized Estimating Equation (GEE) analysis regression coefficients estimated using the maximum likelihood method were used. The resulting model for MD took the following form:

**MD = 0.21· Parafovea deep-0,03·Temporal-0,08·Whole sup.-3.58**

Table 1. The results for MD (GEE)

|  | Assesment | Standard error | Wald’s statistic | Confidence interval +95.0% | Confidence interval  -95.0% | p |
| --- | --- | --- | --- | --- | --- | --- |
|  | -3.583 | 1.662 | 4.648 | -6.840 | -0.326 | 0.03 |
| Parafovea deep | 0.212 | 0.030 | 51.409 | 0.154 | 0.269 | 0.000000 |
| Temporal | -0.031 | 0.006 | 31.620 | -0.042 | -0.020 | 0.000000 |
| Whole sup. | -0.078 | 0.023 | 11.230 | -0.124 | -0.032 | 0.0008 |
| Scale | 1.981 | 0.495 |  | 1.214 | 3.234 |  |

From the obtained results, we conclude that all estimated parameters were highly significant. This is indicated by the Wald statistic and the probability level p related to it for Parafovea deep and Temporal p <0.000001 and Whole sup. p = 0.0008. The increase in the value of Parafovea deep influences the increase of the MD field results, while the increase of Temporal and Whole sup. affects the decrease in MD value.

The maximum likelihood (NW) estimator was 3.58. The unbiased estimator (deviation / df) was 7.85 (table below).

|  | Df | Stat | Stat/Df |
| --- | --- | --- | --- |
| Deviation | 4 | 31.401 | 7.850 |
| Scale of deviation | 4 | 8.000 | 2.000 |
| Chi2 Pearson’s | 4 | 31.401 | 7.850 |
| Skalow. Chi2 P. | 4 | 8.000 | 2.000 |
| Log |  | -16,821 |  |

The resulting model for PSD took the following form:

**PSD = 0.042· Whole deep-0,026·Superior+0,061·Inferior-2.901**

|  | Assesment | Standard error | Walda’s Statistic | Confidence interval  +95.0% | Confidence interval  -95.0% | p |
| --- | --- | --- | --- | --- | --- | --- |
|  | -2.901 | 0.553 | 27.524 | -3.985 | -1.817 | 0.000000 |
| Whole deep | 0.042 | 0.007 | 31.425 | 0.027 | 0.057 | 0.000000 |
| Superior | -0.026 | 0.002 | 166.721 | -0.030 | -0.022 | 0.000000 |
| Inferior | 0.061 | 0.005 | 130.678 | 0.050 | 0.071 | 0.000000 |
| Skala | 0.582 | 0.146 |  | 0.357 | 0.950 |  |

From the obtained results, we conclude that all estimated parameters were highly significant. This is indicated by the Wald statistic and the probability level p with it was highly significant (p <0.000001). An increase in Whole deep and Inferior values ​​increases the PSD field results, while an increase in Superior affects the decrease in MD value.

The maximum likelihood estimator (NW) was 2.901. The unbiased estimator (deviation / df) was 0.678 (table below)

|  | Df | Stat | Stat/Df |
| --- | --- | --- | --- |
| Odchylenie | 4 | 2.711 | 0.678 |
| Skal.odchylenie | 4 | 8.000 | 2.000 |
| Chi2 Pearsona | 4 | 2.711 | 0.678 |
| Skalow. Chi2 P. | 4 | 8.000 | 2.000 |
| Log(il.wiar.) |  | -7,023 |  |

We did not obtain a significant model for isopters.
